# Enhancing the XGBoost Mortality Prediction Model for ICU Patients with Acute Ischemic Stroke

**DOI:** 10.34133/hds.0371

**Published:** 2025-12-09

**Authors:** Jing Sun, Chang Meng, Guobin Miao

**Affiliations:** Department of Emergency and Critical Care Center, Emergency General Hospital, Beijing 100028, PR China.

We value Cummins et al.’s study on an XGBoost model for in-hospital mortality prediction in intensive care unit (ICU) patients with acute ischemic stroke (AIS) [1]. Its focus on model fairness across demographics and in-depth feature analysis gives useful references for clinical predictive analytics. To boost the model’s reproducibility, we suggest clarifying specific inclusion criteria and data preprocessing methods for the electronic ICU (eICU) database in future external validation.

This study has effectively identified key prognostic features including Glasgow coma scale (GCS) scores and blood urea nitrogen (BUN) levels and provided a solid foundation for clinical application. Relevant literature supports the importance of these variables in ICU stroke patients’ mortality risk [2]. To expand the model’s practical value, we propose 3 focused directions. First, integrating real-time electronic health record (EHR) data such as hourly updated vital signs could turn the model from static to dynamic. This would let clinicians adjust interventions in real time to better align with ICU care needs. Second, exploring interactions between top features, such as if low GCS scores paired with high BUN levels increase mortality risk more notably, could provide nuanced risk insights for bedside decision-making. Third, extending predictions to post-discharge outcomes like 30- or 90-d mortality would align with long-term AIS management. If the Medical Information Mart for Intensive Care-IV (MIMIC-IV) database includes follow-up data or links to other hospitals’ records, this extension could help design targeted follow-up plans for high-risk patients and improve their long-term prognosis. These steps would enhance the model’s clinical utility and confirm its stability across diverse care scenarios.
